# Association of Serum Albumin and Severity of Pulmonary Embolism

**DOI:** 10.3390/medicina56010026

**Published:** 2020-01-09

**Authors:** Hesham R. Omar, Mehdi Mirsaeidi, Rania Rashad, Hatem Hassaballa, Garett Enten, Engy Helal, Devanand Mangar, Enrico M. Camporesi

**Affiliations:** 1 Internal Medicine physician at Online Care Group (AmericanWell.com), Chicago, IL 60585, USA; 2 Division of Pulmonary, Critical Care, Sleep and Allergy, University of Miami, Miller School of Medicine, Miami, FL 33136, USA; golmeh@gmail.com; 3 Section of Pulmonary, Department of Medicine, Miami VA Medical Center, Miami, FL 33136, USA; 4 Division of Critical Care Medicine, Alain Hospital, Al Ain 15258, UAE; Rania185@hotmail.com; 5 Swedish American Hospital, Rockford, IL 61104, USA; hatem.hassaballa@gmail.com; 6 Tampa General Hospital, Tampa, FL 33606, USA; garrett.enten@gmail.com; 7 Department of Public Health, Loyola University Chicago, Chicago, IL 60153, USA; ehelal@luc.edu; 8 Department of Anesthesiology, Tampa General Hospital, Tampa, FL 33606, USA; dmangar1@gmail.com; 9 TEAMHealth, Tampa, FL 33606, USA; 10 Departments of Surgery/Anesthesiology, Molecular Pharmacology and Physiology, University of South Florida, FGTBA and TEAMHealth, Tampa, FL 33606, USA; ecampore@health.usf.edu

**Keywords:** inflammation, pulmonary embolism, albumin, red cell distribution width

## Abstract

*Background and Objectives:* Inflammation is considered a risk factor for venous thromboembolism. The association between inflammatory markers and the severity of acute pulmonary embolism (APE) has not been explored. *Methods:* We studied the association between two crude markers of inflammation, serum albumin, and red cell distribution width (RDW) and massive versus non-massive APE. *Results:* Among 552 consecutive cases of CT-angiogram-confirmed APE, a total of 46 cases (8.3%) had massive APE. Despite similar demographics and comorbidities, patients with massive APE had higher frequency of acute kidney injury (*P* = 0.005), higher lactic acid (*P* = 0.011), higher troponin (*P* = 0.001), higher BNP (*P* < 0.001), higher frequency of RV dilation (*P* = 0.017) and hypokinesis (*P* = 0.003), and higher in-hospital mortality (15.2% vs. 2%, *P* < 0.001). Patients with massive APE had significantly lower albumin level (median (IQR): 2.8 (2.2, 3.0) vs. 3.2 (2.8, 3.6) gm/dL, *P* < 0.001) and higher RDW (median (IQR): 14.7 (13.8, 17.1) vs. 14.2 (13.3, 15.6), *P* = 0.006) compared with non-massive APE. ROC curves showed that albumin and RDW had an AUC of 0.750 (*P* < 0.001) and 0.621 (*P* = 0.006) in predicting a massive APE, respectively. The optimal cutoff values for albumin and RDW that had the highest combined sensitivity and specificity for predicting APE was ≤3 gm/dL and >14, for albumin and RDW, respectively. Restricted cubic splines showed a significant association between albumin (*P* = 0.0002) and RDW (*P* = 0.0446) and the occurrence of massive APE. After adjustment for patients’ age, body mass index, white blood cell count, the requirement of antibiotics during hospitalization, diabetes, RDW, and peak creatinine, serum albumin was independently associated with massive APE (OR 0.234, 95% CI 0.129–0.4242, *P* < 0.001). *Conclusion:* low serum albumin is associated with massive APE. This association is likely a proxy for higher inflammatory state in massive compared with non-massive APE.

## 1. Introduction

There is an elevated risk of venous thromboembolism in chronic inflammatory conditions such as vasculitis, sarcoidosis, rheumatoid arthritis, inflammatory myopathies, celiac disease, and inflammatory bowel disease [1,2,3], as well as an up-regulation of inflammatory mediators during acute thrombosis. The modulation of inflammation with antiplatelets [4] and statins [5] was found to reduce the risk of thrombotic events. To the contrary of previous belief that venous thromboembolism is pathophysiologically distinct from atherothrombotic disorders, it is now suggested that it should be considered a part of a “pan-cardiovascular syndrome” [6] that includes coronary artery disease, peripheral arterial disease and cerebrovascular disease [7], as they all share the same underlying hypercoagulability, endothelial injury as well as inflammation [8]. 

At the onset of acute pulmonary embolism (APE), there are almost no inflammatory cells in the walls of pulmonary arteries. Over the next three hours and peaking at two days there is an incremental increase in leucocyte infiltration of the pulmonary arterial wall, which is followed by a considerable reduction of inflammatory cell infiltration after four days and returning to baseline after eight days [9]. 

Several studies confirmed the inflammatory response in APE suggesting the potential value of inflammatory markers in the diagnosis and prognosis in such cases. C-reactive protein, a well-known marker of inflammation and tissue damage, was extensively studied in venous thromboembolism and had a sensitivity ranging 60% and 100% at the level between 5 mg/L and 10 mg/L and specificity varying between 52% and 78% [10,11,12,13,14,15]. Several studies reported that CRP had a sensitivity of 100% for excluding APE [13,14]. Among the 10,505 participants in the ARIC (Atherosclerosis Risk in Community) who were followed for incident deep venous thrombosis or APE over a period of 8.3 years, an increase in CRP concentration above the 90th percentile was associated with a 76% increased risk of venous thromboembolism [16]. It was also found to have a prognostic value where it is associated with right ventricular (RV) dysfunction, a predictor of worse outcomes in APE [17]. Other studies showed an increase of leucocytes and other inflammatory markers including erythrocyte sedimentation rate, and interleukin 6 [18,19] and red cell distribution width (RDW) [20]. 

Elevated RDW indicates a greater difference in RBCs size and was found to have a graded association with CRP and ESR independent of confounding factors [20]. Also, low serum albumin was associated with increased risk of venous thromboembolism in several renal pathologies associated with proteinuria [21,22], although some studies have not found this association [23,24,25,26]. An explanation for such association is either that hypoalbuminemia may be a marker of inflammation as albumin is a negative acute phase reactant, or it is a reflection of renal loss of albumin and anti-thrombotic proteins creating a hypercoagulable state. Folsom and colleagues investigated the association between albumin and incident venous thromboembolism in the general population from two large prospective population-based cohorts: the ARIC study (*n* = 15,300) and the Cardiovascular Health Study (*n* = 5400), and concluded that low serum albumin is a marker of venous thromboembolism risk [27]. 

The degree of inflammation according to severity of APE has not been previously studied. We hypothesized that there is a higher degree of inflammation in massive versus non-massive APE and utilized two crude markers of inflammation to investigate this hypothesis: albumin and RDW.

## 2. Methods

This is a retrospective analysis of consecutive adult patients (>18 years of age) with an International Classification of Disease (ICD-10-CM) diagnosis of computed tomography pulmonary angiogram (CTA)-confirmed APE admitted to Tampa General Hospital between 2010–2015. Patients’ demographics, comorbidities, presenting symptoms, laboratory tests, echocardiography, medications, interventions, and outcomes were collected. The Institutional Review Board at Tampa General Hospital approved the study and waived the need for patient consent.

To investigate a research question set a priori to further study the role of inflammation in APE we have collected data on two markers of inflammation present in basic laboratory workup: Albumin which is a negative acute phase reactant (so lower level is associated with a higher degree of inflammation) and RDW (higher level is associated with a higher degree of inflammation). We aim to identify differences in the degree of inflammation in massive versus non-massive APE. Massive APE was defined as pulmonary embolism associated with systolic blood pressure (SBP) < 90 mmHg or requiring inotropic support [28]. Meeting criteria of massive APE was adjudicated by the study investigators after chart review considering the prespecified above mentioned definitions. All other cases were included with the “non-massive APE” group. RV dysfunction was diagnosed in our study with the presence of one of the following: Right ventricular dilation or hypokinesis on echocardiography or elevated B-type natriuretic peptide (BNP), and myocardial necrosis was defined by elevation of cardiac troponin I > 0.4 ng/mL [28]. Acute kidney injury was defined as a rise in serum creatinine of 0.3 mg/dL or >50% increase from the baseline value.

### Statistical Analysis

Primary analysis compared patients hospitalized with massive versus non-massive APE. The Shapiro–Wilk test was used to assess normality of distribution of continuous variables. Because the majority of variables were not normally distributed, the Mann–Whitney U test was used for comparison of continuous variables which were listed as median and interquartile range (IQR). The chi-square test was used to compare both groups on categorical variables, which were then summarized using counts and percentages. Occasionally, an estimated odds ratio and 95% confidence interval were calculated using the Mantel-Haenszel common odds ratio estimation. The ability of levels of albumin and RDW to predict a massive APE was assessed by calculating the area under the curve (AUC) of the receiver operating characteristic (ROC) curve. Restricted cubic splines were used to assess the unadjusted relationships between albumin, RDW and massive APE. A multivariate logistic regression analysis was performed to identify independent predictors of the occurrence of “massive APE”. The outcome variable was massive APE and the predictor variables included those that had a *P* < 0.05 upon univariate analysis or those considered to be clinically relevant. The goodness-of-fit of the model was examined by the Hosmer–Lemeshow test. Statistical analysis was performed using IBM SPSS 21.0 statistical software (IBM SPSS Version 21.0, Armonk, NY, USA). All statistical significance was assessed using a 2-sided *P* values. A *P*-value < 0.05 was considered statistically significant.

## 3. Results

### 3.1. Patient Characteristics

A total of 552 subjects had CTA-confirmed APE among which 46 (8.3%) had massive APE. The mean age of the study population was 54 ± 17 years, 47% (260/552) were men, 28% (154/552) were African Americans, 59% (326/552) were European Americans, and 21% (118/552) had prior history of APE. On hospital admission, 37% of patients (202/550) were on aspirin, 8% (44/550) were on clopidogrel, and 42% (233/552) were already on anticoagulant medications. Thirty-five percent (191/552) of the subjects had concomitant acute deep venous thrombosis. All subjects were anticoagulated with either intravenous unfractionated heparin, low molecular weight heparin, or novel anticoagulants. 

Despite no difference in baseline demographics and comorbidities, patients with massive APE had a higher frequency of acute kidney injury (*P* = 0.005) and a higher lactic acid (*P* = 0.011), cardiac troponin I (*P* = 0.001) and B-type natriuretic peptide (*P* < 0.001), a higher frequency of RV dilation (*P* = 0.017) and hypokinesis (*P* = 0.003), higher requirement of mechanical ventilation (*P* < 0.001) and higher in-hospital mortality (15.2% vs. 2%, *P* < 0.001). Detailed comparison of subjects with massive versus non-massive APE is listed in Table 1.

### 3.2. Albumin and RDW in Massive Versus Non-Massive Pulmonary Embolism

**Figure 1:**
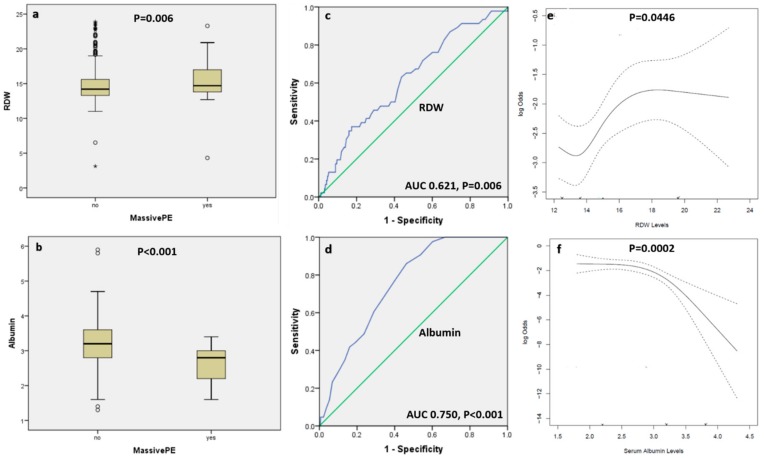
Box and whisker plots showing the level of red cell distribution width (panel **a**) and albumin (panel **b**) among patients with massive versus non-massive acute pulmonary embolism. The five horizontal lines represent the 10th, 25th, 50th, 75th, and 90th percentiles of each variable, from bottom to top, excluding outliers which are shown as circles and extreme outliers shown as asterisk. Panels **c** and **d** are receiver operator characteristic curves showing the area under the curve for RDW and albumin in predicting massive pulmonary embolism, respectively. Panels **e** and **f** are restricted cubic splines which depict the unadjusted associations between RDW and albumin and massive pulmonary embolism, respectively. The dotted curves represent pointwise 95% confidence bands. The *p*-values for the spline models, and the fact that a horizontal line can’t fit within the confidence bands, favors the association.

Patients with massive APE had a significantly lower albumin level [median (IQR): 2.8 (2.2, 3) vs. 3.2 (2.8, 3.6) gm/dL, *P* < 0.001) and higher RDW (median (IQR): 14.7 (13.8, 17.1) vs. 14.2 (13.3, 15.6), *P* = 0.006) compared with subjects with non-massive APE, respectively (Figure 1 panel a, b). Receiver operator characteristics curve showed that albumin had an area under curve (AUC) of 0.750 (95% CI 0.690–0.810, *P* < 0.001), and RDW had an AUC of 0.621 (95% CI 0.539–0.704, *P* = 0.006) in predicting a massive APE (Figure 1 panel c, d). The optimal cutoff values for albumin and RDW that had the highest combined sensitivity and specificity for predicting APE was ≤3 gm/dL for albumin (77% sensitivity and 61% specificity) and >14 for RDW (72% sensitivity and 46% specificity), respectively. Using restricted cubic splines to examine unadjusted relationships between albumin and RDW, and massive APE, we found a significant association between albumin (*P* = 0.0002) and RDW (*P* = 0.0446) and the occurrence of massive APE (Figure 1, panel e, f).

### 3.3. Multivariable Analysis of Factors on Admission Associated with Massive APE

In the multivariate logistic regression model (N = 507, *P* from Hosmer and Lemeshow = 0.433), we found that albumin level on admission independently predicted a massive APE (OR 0.234, 95% CI 0.129–0.4242, *P* < 0.001) after adjusting for patients’ age, body mass index, peak creatinine level, white blood cell count, requirement of antibiotics during hospitalization, the presence of diabetes mellitus and RDW. Results of variables included in the multivariate model is listed in Table 2. 

## 4. Discussion

We have shown in this analysis the association between serum albumin and severity of APE. For every 1 gm/dL reduction in albumin level, a massive APE was 75% more likely. The findings remained unaltered despite multivariable analysis adjusting for multiple confounders including BMI, a rough marker of malnutrition, WBC count a rough marker of acute infectious process, the presence of diabetes which is known to be associated with lower albumin level [29] and serum creatinine because of the reports linking albuminuria and venous thromboembolism. These reports were mainly in patients with nephrotic syndrome which is a rare entity and is unlikely to explain our observations. Even in chronic kidney disease serum albumin is usually unaffected except in late stages [30] which was also rare in our cohort. Moreover, the association between hypoalbuminemia and venous thromboembolism in renal conditions specifically nephrotic syndrome is mainly explained by the renal loss of antithrombotic proteins creating a state of hypercoagulability and hence this cannot be a plausible explanation for the association we find in the general population. Several retrospective studies confirmed the association between lower albumin and venous thromboembolism in the general population without nephrotic syndrome [31], in addition, one prospective study from an analysis of the ARIC and CHS [27] where albumin was measured before rather than after the thrombotic event also confirmed this association. This is the first study to link albumin level with severity of APE (massive versus non-massive cases) with big effect size between groups (2.8 gm/dL and 3.2 gm/dL in massive and non-massive APE, respectively) and hence it serves not only as a diagnostic but also a prognostic marker. 

This association between albumin and APE severity does not reflect a cause and effect relationship but is rather a proxy for underlying inflammation. There is still curiosity about whether inflammation is a cause or a consequence of the thrombotic process, nonetheless, most of the evidence favors that inflammation is a direct cause of venous thromboembolism rather than a consequence [8,32]. A low albumin can be an indicator of poor general health and malnutrition, however, this is less likely to explain our findings since as both groups in our cohort had a comparable BMI of ~30 Kg/m^2^. It was shown that in chronic inflammatory conditions, hypoalbuminemia is a marker of inflammation rather than malnutrition. We have recently reported the association of low serum albumin with the degree of inflammation rather than malnutrition in patients with pulmonary sarcoidosis [33]. In subjects with pulmonary sarcoidosis, a significant inverse correlation was found between albumin and ESR (*r* = −0.630, *P* = 0.0001), and CRP (*r* = −0.350, *P* = 0.001) but no relationship between albumin and BMI, a crude marker used for nutritional status (*r* = 0.015, *P* = 0.574).

### Study Limitations

There are several limitations in our study, mainly those inherent to non-randomized trials. It is a retrospective analysis with a relatively small sample size in the study arm with massive APE. The tested markers of inflammation (albumin and RDW) were measured on hospital admission, and one may assume that, at least for the massive APE group (8.3% of the study population), the derangement of these laboratory parameters may be caused by shock and hemodynamic instability on presentation. The erythrocyte sedimentation rate and C-reactive protein were collected for approximately 50 patients only and so we could not further confirm the association with lower albumin and a hyperinflammatory state. Serum albumin was measured at hospital admission after the diagnosis of pulmonary embolism so it is hard to differentiate whether hypoalbuminemia is a cause or a consequence of the thromboembolic process. There are probably other confounding variables that affect albumin level and were not accounted for and may have altered the results such as chronic inflammatory conditions and liver disease.

## 5. Conclusions

In conclusion, our findings support an association between a lower albumin level and a more severe APE. We propose the need for further prospective studies to see if chronic hypoalbuminemia can identify those at risk of thromboembolism and the role of anti-inflammatory drugs in secondary prevention of APE. 

## Figures and Tables

**Table 1:** Comparison of characteristics of patients with versus without massive pulmonary embolism.

	Massive APE (*n* = 46)	Non-Massive APE (*n* = 506)	*P*-Value
**Demographics**			
Age (y, median, IQR)	58 (42, 69)	54 (42, 66)	0.391
BMI (Kg/m^2^, median, IQR)	30.3 (25.6, 38.4)	29.8 (25.2, 36.7)	0.386
Men % (n)	56.5% (26/46)	46.2% (234/506)	0.181
Black % (n)	32.6% (15/46)	27.5% (139/506)	0.457
White % (n)	50% (23/46)	59.9% (303/506)	0.192
Smoking % (n)	26.1% (12/46)	26.3% (133/506)	0.977
**Comorbidities**			
DM % (n)	30.4% (14/46)	31.2% (158/506)	0.912
COPD % (n)	10.9% (5/46)	14.8% (75/506)	0.466
History of cancer % (n)	32.6% (15/46)	27.5% (139/506)	0.457
SLE % (n)	4.3% (2/46)	2.6% (13/506)	0.477
Prior DVT % (n)	28.3% (13/46)	23.9% (121/506)	0.510
Prior PE % (n)	28.3% (13/46)	20.8% (105/506)	0.234
Orthopedic fracture in past 90 d % (n)	23.9% (11/46)	13.8% (70/506)	0.064
**Clinical features**			
SBP < 90 mmHg % (n)	52.2% (24/46)	0% (0/506)	<0.001
HR > 100 bpm % (n)	52.2% (24/46)	29.8% (151/506)	0.002
SO2 < 88% on RA % (n)	17.8% (8/45)	3.4% (17/503)	<0.001
Pressor support % (n)	58.7% (27/46)	0% (0/506)	<0.001
New onset AFib % (n)	8.7% (4/46)	6.1% (31/506)	0.494
Antibiotic requirement % (n)	54.3% (25/46)	34.8% (176/506)	0.008
consolidation on CT scan % (n)	17.8% (8/45)	12.4% (60/483)	0.305
Acute kidney injury % (n)	20% (9/45)	7.6% (37/487)	0.005
**Laboratory**			
AST (IU/L, median, IQR)	26 (20, 48)	25 (20, 34)	0.177
ALT (IU/L, median, IQR)	22 (16, 44)	23 (16, 33)	0.453
Peak creatinine (mg/dL, median, IQR)	1 (0.8, 1.8)	0.9 (0.8, 1.2)	0.036
Lactic acid (mmol/L, median, IQR)	2.5 (1.3, 5)	1.3 (0.9, 2)	0.011
Troponin (ng/mL, median, IQR)	0.27 (0.05, 0.62)	0.05 (0.01, 0.44)	0.001
BNP (pg/dL, median, IQR)	357 (82, 786)	25 (11, 127)	<0.001
Albumin (g/dL, median, IQR)	2.8 (2.2, 3)	3.2 (2.8, 3.6)	<0.001
RDW (median, IQR)	14.7 (13.8, 17.1)	14.2 (13.3, 15.6)	0.006
WBC /microliter (median, IQR)	9.2 (6.6, 12.6)	8.8 (6.5, 11.4)	0.382
**Echocardiography**			
RV dilation (echo) % (n)	41.7% (15/36)	23.4% (70/299)	0.017
RV hypokinesis (echo) % (n)	38.9% (14/36)	17.7% (53/299)	0.003
**Treatment**			
Mechanical ventilation % (n)	21.7% (10/46)	2.8% (14/506)	<0.001
Systemic thrombolysis % (n)	17.4% (8/46)	3.8% (19/506)	<0.001
**Outcomes**			
Hospital duration (d, mean, SD)	0.84 ± 0.84	0.4 ± 0.59	0.001
Survived hospitalization % (n)	84.8% (39/46)	98% (496/506)	<0.001

BMI: body mass index, DM: Diabetes Mellitus, COPD: chronic obstructive pulmonary disease, SLE: systemic lupus erythematosus, DVT: deep venous thrombosis, PE: pulmonary embolism, SBP: systolic blood pressure, HR: heart rate, SO2: oxygen saturation, RA: room air, RR: respiratory rate, BNP: B-type natriuretic peptide, RDW: red cell distribution width, RV: right ventricle, BiPAP: bilevel positive pressure ventilation.

**Table 2:** Multivariate analysis of determinants of massive acute pulmonary embolism.

	OR; 95% CI; *P*-Value
Age	1.014 (0.993, 1.035) 0.185
BMI	1.030 (0.996, 1.065) 0.087
Albumin	0.234 (0.129, 0.4242) <0.001
RDW	0.985 (0.870, 1.139) 0.942
DM	0.996 (0.475, 2.026) 0.991
WBC	1.000 (0.968, 1.033) 0.994
Antibiotic requirement	1.600 (0.810, 3.162) 0.176
Peak creatinine	1.145 (1.009, 1.299) 0.036

BMI: body mass index, RDW: red cell distribution width, DM: Diabetes Mellitus, WBC: white blood cell count.
